# Infection control link nurse programs in Dutch acute care hospitals; a mixed-methods study

**DOI:** 10.1186/s13756-020-0704-2

**Published:** 2020-02-27

**Authors:** Mireille Dekker, Rosa van Mansfeld, Christina Vandenbroucke-Grauls, Martine de Bruijne, Irene Jongerden

**Affiliations:** 1 Department of Medical Microbiology and Infection Prevention, Amsterdam UMC, Vrije Universiteit Amsterdam, De Boelelaan 1118, room PK1X132, 1081 HV Amsterdam, The Netherlands; 2 Department of Public and Occupational Health, Amsterdam Public Health research institute, Amsterdam UMC, Vrije Universiteit Amsterdam, Amsterdam, The Netherlands

**Keywords:** Liaison nurse, Infection prevention and control, Nosocomial infections, Cross infection, Social sciences, Multi-modal intervention, Compliance, Infection control guidelines, Guideline adherence

## Abstract

**Background:**

Infection control link nurse programs show considerable variation. We report how Dutch link nurse programs are organized, how they progress, and how contextual factors may play a role in the execution of these programs.

**Methods:**

This mixed-methods study combined a survey and semi-structured interviews with infection control practitioners, based on items of the Template for Intervention Description and Replication (TIDieR) checklist.

**Results:**

The Netherlands has 74 hospitals; 72 infection control practitioners from 72 different hospitals participated in the survey. Four of these infection control practitioners participated in interviews. A link nurse program was present in 67% of the hospitals; responsibility for 76% of these programs lied solely with the infection prevention and control team. The core component of most programs (90%) was education. Programs that included education on infection prevention topics and training in implementation skills were perceived as more effective than programs without such education or programs where education included only infection prevention topics.

The interviews illustrated that these programs were initiated by the infection prevention team with the intention to collaborate with other departments to improve practice. Content for these programs was created at the time of their implementation. Infection control practitioners varied in their ability to express program goals and to engage experts and key stakeholders.

**Conclusions:**

Infection control link nurse programs vary in content and in set up. Programs with a clear educational content are viewed as more successful by the infection control practitioners that implement these programs.

## Introduction

Healthcare-associated infections are the most frequent adverse event for patients admitted to hospitals, and an important cause of morbidity and mortality [1, 2]. Careful infection prevention and control (IPC) measures can prevent up to a third of these infections [3]. IPC measures are laid down in guidelines and policies at the national and international level [2, 3]. Implementation of these guidelines is usually the task of infection prevention and control teams. In many Dutch hospitals these teams are supported by infection control link nurses (ICLN) [4]. In all countries were ICLN have been introduced, these nurses act as a link between colleagues in their own clinical area and the infection prevention and control team, and help raising awareness of infection control by educating colleagues and motivating staff to improve practice [4, 5].

Review of the literature on ICLN show that link nurse programs have been implemented all over the world. The majority of this literature originates from the United Kingdom and describes variation in how ICLN programs are organized and implemented [6]. This variation relates to all aspects of such programs - i.e. responsibilities and tasks of ICLN, activities for and education of ICLN, and competences that are required to fulfill the ICLN role [6–8]. The few studies that have evaluated effectiveness of these programs revealed that compliance with hand hygiene guidelines and incidence of MRSA infections indeed improve when ICLN are active [9, 10]. However, these studies do not describe their ICLN program in detail nor elaborate on the contextual factors that may have contributed to these improvements. Contextual factors include factors that are not part of the ICLN program such as cultural, organisational and management characteristics of the hosptial, but do play a role in the implementation of IPC practices [11, 12]. Examining the variation of existing ICLN programs, the assessment of contextual factors that have led to this variation and the evaluation of these programs can reveal opportunities to improve their value and to reduce their inefficiencies. We therefore aimed to describe how Dutch ICLN programs are organized and how they progress. Furthermore, we sought to explore the contextual factors that may have influenced the implementation of these programs.

## Methods

### Study design

In a mixed-method study, we combined a cross-sectional survey with additional semi-structured interviews, based on items of the Template for Intervention Description and Replication (TIDieR) checklist [13]. The TIDieR checklist is an extension of the CONSORT 2010 and SPIRIT 2013 statement and was designed to guide the description of trial interventions in sufficient detail to allow their replication. It has proven to be also applicable for reporting and evaluation of complex interventions in non-trial settings [14, 15]. The checklist consists of items concerning: the name of the intervention, the rationale, theory or goal of intervention elements, procedures; providers; how the intervention was delivered and where; the number of times the intervention was delivered and over what period of time if it was tailored, adapted or modified; and if fidelity was assessed.

To describe the Dutch ICLN programs we developed a survey. Survey questions were based on recent literature on ICLN and categorized according to the TIDieR checklist items [6, 16, 17]. The survey contained multiple choice questions, some with multiple answer options. Three infection control practitioners and an epidemiologist pilot tested the survey. After adjustments it was divided in five parts. The first part contained questions on the presence of an ICLN program or the intention to set up such a program. The second part zoomed in on tasks, goals, and activities of the link nurses. In the third part, infection control practitioners were asked which competences they consider important to fulfill the ICLN role. The fourth part covered the educational content and the evaluation of the program. In the final part, respondents were asked to what extent they were able to accomplish their IPC goals through the help of ICLN. This was expressed on a 10 point Likert scale.

Cotterill et al. recommended to describe how contextual factors may have influenced the execution of the intervention to compile a more realistic image of implementation in real life practice, and proposed to extend the TIDieR checklist by four items [18]. These items include the incorporation of the perspectives of those who provided the intervention, the stage of implementation (e.g. from proof of concept to long term sustainability) the intervention has reached, a description of adaptations made to any item in the checklist, and an outline of factors which had impact on how the intervention was implemented.

To explore how contextual factors had influenced implementation and to investigate the real life practice of ICLN programs, selected infection control practitioners were interviewed in a semi-structured way. The interviews allowed the additional exploration of personal views, experiences and perceptions on why and how specific components of the ICLN program were chosen, how the program was realized in practice, and how it changed over time [19, 20]. A topic list (Table 1) based on the checklist extensions as described by Cotterill et al., guided the face to face interviews.

**Table 1:** Topic list

Topic list	
1. Delineation of the ICLN program o the start o the goals ■ what are the goals? ■ what actions are necessary to achieve goals? ■ how do you know if you have achieved a goal? ■ what helps in achieving goals? ■ what does not help? o the plan ■ where adjustments made to the plan? ■ how would you know if adjustments are necessary? 2. Embedding of the program ■ how do you secure continuity and effectiveness? ■ what is the role of the infection control practitioners? ■ what is the role of others?	

### Data collection

During a National Congress for Dutch infection control practitioners in April 2018, surveys were distributed to and collected from one infection control practitioner per Dutch hospital (*n* = 74) with inpatient departments. One week after the congress, infection control practitioners who did not return their survey were contacted by telephone. To further explore survey answers, we conducted semi-structured interviews with infection control practitioners between July 2018 and October 2018. To explore multiple perspectives a purposeful sampling technique was applied [20, 21]. Selection of infection control practitioners was based on the duration of the program in their hospital and how the practitioner graded the effects of the program. The interviews were conducted by one researcher (MD). Interviewees were informed about the study goals, and that there were no right or wrong answers. They were assured anonymity and provided a written consent. The results of the interviews are reported according to the Consolidated Criteria for Reporting Qualitative Research checklist [22].

### Data analysis

Surveys and interviews were analysed separately. Subsequently, survey and interview outcomes were compared to integrate the findings [23].

Surveys were included in the analysis if ≥50% of questions were answered. Survey data were analysed using descriptive statistics. Items that were identified as best practices in ICLN programs in previous studies were compared [6]. These best practices are the availability of a written role profile, education on infection prevention topics as well as on implementation skills, and support of ICLN by the ward manager. Differences in median values for the achievement of program goals between groups were analysed with the Mann-Whitney U test for comparison of two groups and the Kruskal-Wallis test for comparison of three groups. A post-hoc test was performed with a Kruskal-Wallis test with Bonferroni correction for a pairwise comparison of the educational programs. A boxplot was created based on this comparison. Analyses were performed with R Studio version 5.0–0 (R Foundation for Statistical Computing, Vienna, Austria).

Interviews were audio recorded, transcribed verbatim (MD) and analysed by thematic analyses with an iterative, inductive approach [24, 25]. Two team members (MD & RM) read the transcripts several times and independently coded the transcripts to reflect the underlying meaning of the text. Codes were compared and discussed to reach consensus on code names and meaning (MD, RM & IJ). A codebook was created. These codes were clustered into categories and ultimately into themes. During team meetings the influence of the researchers’ backgrounds (Public and Occupational Health, Clinical Microbiology, and Infection Control) was reflected on to further enhance research rigor [26]. Transcripts were analysed with Atlas. Ti software version 7.0 for Windows.

### Results

In total, 72 of 74 questionnaires were returned (response rate 97.3%) (Supplementary materials 1). Forty-eight (66.7%) came from hospitals with an ICLN program in place. Eighteen (25%) came from hospitals that were planning to implement such a program in the near future. Six (8.3%) reported the ceasing of their link nurse program due to lack of support from ward and hospital management (*n* = 2), lack of time and power that was allotted to ICLN (*n* = 3), or other hospital priorities (merger) (*n* = 1). Nine Dutch synonyms were found for these programs. Participants completed all questions in 47 (65.7%) of 72 surveys. Each participant completed 50% or more of the questions; all surveys were included in the analysis. Four infection control practitioners were interviewed. Duration of the programs in these hospitals ranged from three to 8 years. The interviewees graded the accomplishment of their goals thanks to the help of ICLN as four (*n* = 1), six (*n* = 2), and eight (n = 1) on the 10-point Likert scale. The interviews lasted between 42 and 54 min.

From 523 initial codes, 62 categories and ultimately six themes were identified, four of these were linked to the survey results (Table 2). Quotations are included for illustration.

**Table 2:** Survey results (*n* = 48^a^)

	Proportion (%)
	* Median (IQR)
Characteristics of ICLN programs	
Goals for the program and link nurses	
Increase awareness for infection prevention	46/48 (95.8)
Act as a role model and opinion leader	39/48 (81.3)
Disseminate knowledge on infection prevention	43/48 (89.6)
Act as a source of information for peers	44/48 (91.7)
Contribute to development of ward based infection prevention guidelines	24/48 (50)
Implement guidelines or improve adherence	40/48 (83.3)
Liaise between ward and infection prevention and control team	45/48 (93.8)
Qualities for link nurses to achieve program goals	
Enthusiastic	17/40 (42.5)
Motivated	33/40 (82.5)
Assertive	3/40 (7.5)
Persistent	6/40 (15)
Proactive	28/40 (70)
Natural leader	4/40 (10)
Approachable	15/40 (37.5)
Resilient	4/40 (10)
Responsible	15/40 (37.5)
Respectful	2/40 (5)
Preparation of ICLN programs	
Mode of selection of link nurses	
Nominated by the ward management	32/48 (66.7)
Designated by the ward management	29/48 (60.4)
Approached and invited by the infection prevention and control team	10/48 (20.8)
Voluntary registration	19/48 (39.6)
Recruited though an application procedure	1/48 (2.1)
Other modes of selection	2/48 (4.2)
Health Care Workers involved	
Nurses	47/48 (97.9)
Physicians	1/48 (2.1)
Other HCW (e.g. surgical assistants, physiotherapists, laboratory technicians)	30/48 (62.5)
Departments involved	
Inpatients Wards	47/48 (97.9)
Outpatients Clinics	36/48 (75)
Diagnostics – Day care	38/48 (79.2)
Other departments (e.g. laboratories, operating theatre, facility services)	30/48 (62.5)
Education of ICLN	
Educational program (yes)	42/48 (87.5)
Number of training sessions and meetings per year	
< 4	20/40 (50)
4	14/40 (35)
5	4/40 (10)
6	2/40 (5)
Duration of training sessions or meetings (in hours)	2 (1.4–3.3) *
Modes of education	
Introduction course	
provided by an external party	6/42 (14.3)
an in-house introduction program	24/42 (50)
e-learning	4/42 (9.5)
Regular training/education	
lectures	32/42 (76.2)
skills training	21/42 (50)
simulation based learning	3/42 (7.1)
hospital tours and visits	8/42 (19)
brainstorm sessions	11/42 (26.2)
group discussion/meeting	27/42 (64.3)
teambuilding sessions	3/42 (7.1)
Training and education of link nurses	
Developed by the infection prevention and control team	32/40 (80)
Developed in collaboration with experts (e.g. microbiologists, education experts)	8/40 (20)
Topics for training and education	
Selected by the infection prevention and control team	14/38 (36.8)
Determined by link nurses and the infection prevention and control team	23/38 (60.5)
Topics for education and training	
Planned out in an annual plan	7/35 (20)
Depend on occurring events	28/35 (80)
Responsible for the link nurse program	
Mainly one infection control practitioner	23/45 (51.1)
The infection prevention and control team	11/45 (24.4)
Share the responsibility with other departments	17/44 (38.6)
Evaluation of ICLN programs	
Evaluation	23/45 (51.1)
Based on	
- satisfaction with the program by link nurses and other stakeholders	15/22 (68.2)
- compliance with guidelines in relation to the activities of the link nurses	6/23 (26.1)
- prevalence of Nosocomial infections in relation to the activities of the link nurses	2/23 (8.7)
- other	6/23 (26.1)
Effects of Infection control link nurse programs	
No effect	2/20 (10)
Positive effects	17/20 (85)
Positive and negative effects	1/20 (5)

^a^ not every question was answered by all respondents, therefore denominators vary

The * is specified in the headig of the table = MEDIAN (iqr)

#### The start of ICLN programs

In all hospitals where the infection control team initiated an ICLN program, the initiative for the program originated from their need to collaborate with other departments in the hospital, and from the need to disseminate practical IPC knowledge. The actual start of these programs was related to a more positive overall attitude of hospital management and health care workers towards IPC; it was sparked by threats such as a recent Ebola outbreak and the rise of antimicrobial resistance. The occurrence of outbreaks of resistant strains in hospitals, and pressure from external bodies (e.g. Joint Commission International) increased the urge for hospital management to address IPC as an integral part of patient safety and quality of care. It created opportunities for support for infection control practitioners to start an ICLN program.

*we needed this outbreak of vancomycin-resistant enterococci to convince our hospital management that we needed to implement an ICLN program [interview 4]*

In the first phase of setting up a program, the infection control practitioners pitched and discussed their ideas with middle and higher management.

*I have been to all wards and talked to the management … we were preparing our hospital for a JCI accreditation [interview 1]*

#### The characteristics of ICLN programs

Infection control practitioners aimed to build a structural relationship with the link nurses in order to exchange information on IPC practices and to improve compliance with IPC protocols.

*I hope to learn each link nurse to detect potential infection prevention risks …that they will contact me when they have detected a risk or when they have an IPC related question... I want to team up with these nurses [interview 4]*

The top three goals of ICLN programs were to increase awareness for infection prevention, to create a liaison between the wards and the IPC team, and to make ICLN a source of information for their peers. Some infection control practitioners were able to described these program goals in a clear manner and incorporated knowledge and skills from other departments (e.g. quality department, training and education department) to supplement their own and ICLN’ competences whereas others found it challenging to prepare a plan of action.

*as an infection control practitioner I am obliged to support link nurses, but I don't know how to do that best [interview 2]*

To achieve the program goals, the most sought qualities for ICLN were being motivated, proactive, and enthusiastic. Infection control practitioners’ views on the interaction with the ICLN and communication in the context of the ICLN program varied. Some infection control practitioners focused their efforts on providing support for the ICLN in implementing IPC policies, where others focused more on receiving support from the ICLN in monitoring the compliance with IPC measures.

*you need to listen to the needs of your link nurses...I want to serve them and support them to disseminate their knowledge to their peers on the wards [interview 3]*

#### The preparation of ICLN programs

Most ICLN were nominated by the ward management; clinical experience as a health care worker was not considered necessary. Not only nurses were included, in most hospitals other disciplines and departments also participated. In one hospital physicians were involved. Infection control practitioners described that they developed their programs while implementing them at the same time. Programs were adapted as IPC teams searched for an optimum strategy to collaborate with their link nurses to improve practice. Adjustments to the program were based on lessons learned during implementation and the dynamic IPC priorities. Infection control practitioners query what sort of training to provide, what topics to educate on and how to stimulate ICLN to be proactive.

*Our link nurse meetings must become a bit more interactive. We need to ask , what did you learn? What will you do differently tomorrow? What is the next issue you will address? [interview 3]*

#### The education of ICLN

In almost 90% of the hospitals, programs for ICLN included education, given in sessions with a median duration of two hours, at a frequency of one to six sessions per year. Education of ICLN was generally shaped as in-house training and started with an introduction course. Responsibility to achieve the ICLN program goals lied solely with the IPC team in two thirds of the hospitals.

The IPC teams perceived the introduction of ICLN networks and the activities of ICLN as important assets that helped them to achieve their infection control goals. They scored this importance with a median of 7.0 (IQR 6.0–7.0) on a 10-point Likert scale.

**Fig. 1:**
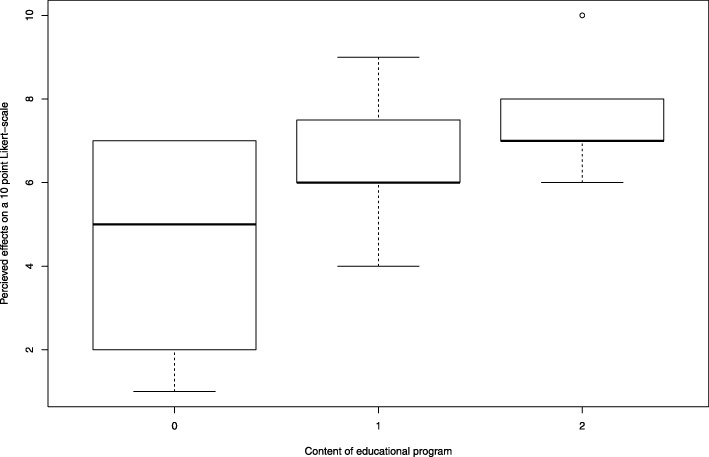
Median perceived effects of educational programs. 0 = no education 1 = education on infection prevention topics 2 = education on infection prevention topics and training in implementation skills

Table 3 displays best practices in ICLN programs and how participants perceived the role of these best-practices in achieving their program goals. In 72% of the hospitals a written role profile was available. The median value for the perceived accomplishment of programs goals for these hospitals did not differ from hospitals that did not provide a written role profile. Seventy-one percent of infection control practitioners reported support from ward management for ICLN in their hospital. The median value for perceived accomplishment of programs goals also did not differ when compared to programs that did not report this support. ICLN programs that included education on infection prevention topics and training in implementation skills were perceived as more effective (median 7.0, IQR 7.0–8.0) than programs without such education (median 5.0, IQR 2.5–6.8) or programs where education included only infection prevention topics (median 6.0, IQR 6.9–7.5) (Table 4) (Fig. 1).

**Table 3:** Comparison of best practices for ICLN programs with perceived accomplishment of program goals

Survey item	Proportion (%)	Perceived accomplishment of program goals (range 1–10) (*n* = 48)	
		Median (IQR)	*p* - value
Written role profile			0.22^a^
Yes	34/47 (72.3)	7.0 (6.0–8.0)	
No	8 /47 (17.4)	6.0 (6.0–8.0)	
don’t know	5/47 (10.6)	6.5 (6.0–8.0)	
Education			0.02^a^
No education	6/48 (12.5)	5.0 (2,5–6.8)	
Education on infection prevention topics	21/48 (43.8)	6.0 (6.0–7.5)	
Education on infection prevention topics and training in implementation skills	21/48 (43.8)	7.0 (7.0–8.0)	
Support			0,09^b^
Support of ICLN by ward management	32/45 (71.1)	7 (6.0–8.0)	
No support of ICLN by ward management	*13/45 (28.9)*	*6 (6.0–7.0)*	

^a^Mann-Whitney U test, ^b^Kruskal-Wallis test

**Table 4:** Comparison of the educational programs with perceived accomplishment of program goals

Education	perceived accomplishment of program goals (range 1–10)	
	Adjusted *p* - value^a^	
	*(0)*	(1)
(0) No education program	–	
(1) Education on infection prevention topic	0.24	–
(2) Education on infection prevention topics as well as training in implementation skills	0.03	0.41

^a^Kruskal-Wallis test with Bonferroni correction for a pairwise comparison

#### The progression of ICLN programs

To better support link nurses with department-specific questions or projects, some infection control practitioners scheduled regular meetings at the department in addition to, or instead of, the hospital wide educational meetings. Furthermore, some infection control practitioners involved ward management in ward-specific ICLN activities to interweave the hierarchical structures with the ICLN program activities. This enabled them to influence both the formal and the informal network to facilitate the program goals and created the opportunity to generate more ward-based support for the ICLN. In parallel, it created an opportunity to increase engagement of other infection control practitioners with the program. Occasionally, meeting attendance by ICLN was registered and reported to the management.

*at the start of this program ICLN educational meetings were mandatory… at that time, we were in the middle of an outbreak, we didn't have enough time to educate our link nurses... nowadays we do not educate in central meetings, we leave it up to the individual IPC team members to maintain intensive contact with their wards and their link nurses. Each Infection control practitioner is responsible for their own contacts and for what is going on in those departments [interview 4]*

Infection control practitioners described the challenge to develop a program that interconnects ICLN of various departments, to create opportunities for ICLN to exchange experiences and ideas. The variation in work environment and training background is considered to cause this lack of interaction between ICLN of different departments.

*we initially wanted to bring link nurses from clinical wards and outpatient clinics together …. during the training it turned out that there was a big difference in knowledge between those two groups…. and that did not correspond so well. They were not able to have meaningful discussions [interview 4]*

The limited time for IC tasks available for link nurses and for ICLN program tasks of the IPC team was mentioned as a barrier to the implementation of ICLN programs.

*last year we could not start the ICLN education for new link nurses …the time was allocated for general education of nurses on the new electronic patient files program [interview3]*

#### The evaluation of ICLN programs

Half of the ICLN programs have been evaluated. Most evaluations (15/22) were based on the satisfaction of stakeholders with the program. Six hospitals evaluated their ICLN program in relation to the adherence to IPC guidelines. Two hospitals evaluated their program in relation to the prevalence of nosocomial infections.

The majority of hospitals that evaluated their program (17/20) reported positive effects. From the interviews arose the impression that these conclusions were based on random observations during ward rounds and gut feeling. Reported effects seemed related to practical issues (e.g. being able to find IPC protocols, stock management of personal protective equipment)

*Link nurses say that we are more visible ... they know how to find us, they consult us. I think that is positive [interview3]*

*I see more information leaflets on infection prevention topics in wards were a link nurse is active [interview 4]*

## Discussion

This mixed methods study provides a detailed overview of infection control link nurse programs in the Netherlands and gives a broader understanding of the factors that can influence the content of these programs and their implementation in acute care hospitals. It confirms the well-known variation in these programs. In addition, our approach permitted us to quantify this variation, and to find opportunities to reduce inefficiencies and to improve the value of these programs. This, to the best of our knowledge was not done before.

Two thirds of Dutch hospitals have an ICLN program in place. Although programs vary widely, education is a core component of nearly all of these programs. ICLN programs are often set up and led solely by the IPC team. Our survey showed that infection control practitioners were more satisfied with their ICLN program if they were able to incorporate training in implementation skills in their educational program. From the interviews it transpired that infection control practitioners seemed more satisfied if they were able 1) to express a more coherent vision and more long term strategic goals 2) to involve more experts (e.g. educational experts) in the enhancement of their program and 3) to engage more key stakeholders, including management, and their direct colleagues, the IPC team, to create support. These aspects therefore, appear useful to keep in mind when planning improvements of existing ICLN programs or when setting up new programs. Overall, our results emphasize that to improve the ICLN programs, infection control practitioners need to have sufficient skills to select and apply appropriate implementation strategies, and to evaluate these strategies to continuously adapt to the dynamic hospital context. In line with this, Gilmartin and colleagues suggests that infection control practices can indeed improve if implementation strategies are systematically considered and applied [27]. The 2017 Geneva Think Tank, a panel of international experts, concluded that implementation science must be a priority in infection prevention [28]. In agreement with our findings it stresses the importance for infection prevention experts as well as other health care workers (e.g. ICLN) to improve their implementation skills.

Education of the link nurses is seen as the core element of ICLN programs although the effect was not systematically measured. Grol et al. nicely summarized the evidence that shows that the dissemination of research findings or guidelines through education can be helpful to realize simple changes in daily practice [29]. However, to improve IPC guideline adherence behavioral change is a prerequisite and such change requires more complex strategies [29–31]. Considering our findings in the light of recommendations made by the World Health Organization, we suggest that ICLN programs should be designed as multimodal interventions [32].. The multimodal approach includes: (1) a comprehensive plan of education, training and communication, (2) the engagement of hospital and ward management, and (3) audit and feedback [28, 32]. It is also important to understand the potential barriers for the implementation of an ICLN program to fit the program to the local context, and to be able to intervene to remove these barriers [29]. We agree with Cunningham et al., that to engage other stakeholders and to collaborate with direct colleagues can help in preventing vulnerability of the program with respect to sustaining network activities [33]. Audit and feedback is essential to boost implementation of IPC policies and can yield valuable input for the evaluation of effects of and refinements to the ICLN program [32, 34]. Finally, and possibly most importantly, ICLN programs should be considered as an integral component of infection prevention and control programs and not as a self-contained project [32].

A major strength of this study is the high survey response rate. It contributed to the representativeness of our findings. We performed additional interviews to deepen our insight in the findings from the survey. This triangulation reduced the chance of single source bias [35]. Furthermore, the interviews reflect real life strategies used by infection control practitioners to disseminate their knowledge through link nurse programs. A deeper understanding of the structure and characteristics of these programs is vital to further develop well-functioning programs [33].

This study has limitations. As the IPC community in the Netherlands is small, respondents might have chosen to respond in a more positive way than to choose the responses that reflected their true thoughts. This social desirability bias could distort the results in the survey and the interviews [36]. To decrease the chance for this bias we assured participants in the survey and in the interviews their anonymity; we also explicitly made it clear that there were no right or wrong responses [36].

The interviews were performed to ad real world examples from link nurse programs to the survey results; the number of interviews was small and therefore may have only provided a limited number of points of view. We provided interview quotes, to enhance transferability of our findings [37].

A follow-up study using social network analysis could operationalize the social structure and cohesion of ICLN networks, their relevance to the implementation of IPC guidelines and clarify how to improve network-based processes to transfer IPC knowledge and support program goals [38–40].

## Conclusion

Infection control link nurse programs in Dutch hospitals originate from a need to collaborate with, and to disseminate practical IPC knowledge to other departments in the hospital. The start of these programs is related to a more positive overall attitude of hospital management and healthcare workers towards infection prevention and control. Although programs vary widely, education is an overall core component. Efforts to improve the uptake of IPC guidelines through ICLN programs should focus on enhancing infection control practitioners’ and link nurses’ knowledge on implementation science and designing these link nurse programs as multimodal interventions. To evaluate the contribution of ICLN programs to the implementation of IPC guidelines it is necessary to audit the program effects and to perform well-designed effectiveness studies. Social network analysis could contribute to understanding how knowledge on infection control and prevention is transferred best.

## Supplementary information

**Additional file 1.** Response rate

## Data Availability

The dataset generated and analysed during the current study and the coding tree (in Dutch) for the interviews are available from the corresponding author on reasonable request.

## Abbreviations

IPC: Infection prevention and control
ICLN: Infection control link nurses
TIDieR: The Template for Intervention Description and Replication checklist
