# Supplementary material for: Infection control link nurse programs in Dutch acute care hospitals; a mixed-methods study
Source: Antimicrob Resist Infect Control. 2020 Feb 27;9:42. doi: 10.1186/s13756-020-0704-2 (PMC7047353; doi:10.1186/s13756-020-0704-2)
Supplement: Supplementary file 1 — Additional file 1. Response rate [file 13756_2020_704_MOESM1_ESM.docx]

**Supplementary materials I** Response rate

| **Response** | |  | **n (%)** |
| --- | --- | --- | --- |
| **number of Dutch Acute Care hospitals** | |  | **79*** |
| number locations excluded | |  |  |
| - locations without inpatient clinic | |  | 4 |
| - location of research team | |  | 1 |
| locations included | |  | 74 |
|  | |  |  |
| questionnaires filled out | |  | 72 |
| locations covert in questionnaires | |  | 72/74 (97.3%) |
|  |  | | |

* Number of Dutch Acute Care Hospitals according to CBS

<https://www.volksgezondheidenzorg.info/onderwerp/ziekenhuiszorg/cijfers-context/aanbod#node-aantal-instellingen-voor-medisch-specialistische-zorg>
